# Automating classification of osteoarthritis according to Kellgren-Lawrence in the knee using deep learning in an unfiltered adult population

**DOI:** 10.1186/s12891-021-04722-7

**Published:** 2021-10-02

**Authors:** Simon Olsson, Ehsan Akbarian, Anna Lind, Ali Sharif Razavian, Max Gordon

**Affiliations:** Department of Clinical Sciences at Danderyd Hospital, Unit of Orthopedics, Karolinska Institutet, 182 88 Stockholm, Sweden

**Keywords:** Deep learning, artificial intelligence, knee osteoarthritis, radiographs, Kellgren & Lawrence classification

## Abstract

**Background:**

Prevalence for knee osteoarthritis is rising in both Sweden and globally due to increased age and obesity in the population. This has subsequently led to an increasing demand for knee arthroplasties. Correct diagnosis and classification of a knee osteoarthritis (OA) are therefore of a great interest in following-up and planning for either conservative or operative management. Most orthopedic surgeons rely on standard weight bearing radiographs of the knee. Improving the reliability and reproducibility of these interpretations could thus be hugely beneficial. Recently, deep learning which is a form of artificial intelligence (AI), has been showing promising results in interpreting radiographic images. In this study, we aim to evaluate how well an AI can classify the severity of knee OA, using entire image series and not excluding common visual disturbances such as an implant, cast and non-degenerative pathologies.

**Methods:**

We selected 6103 radiographic exams of the knee taken at Danderyd University Hospital between the years 2002-2016 and manually categorized them according to the Kellgren & Lawrence grading scale (KL). We then trained a convolutional neural network (CNN) of ResNet architecture using PyTorch. We evaluated the results against a test set of 300 exams that had been reviewed independently by two senior orthopedic surgeons who settled eventual interobserver disagreements through consensus sessions.

**Results:**

The CNN yielded an overall AUC of more than 0.87 for all KL grades except KL grade 2, which yielded an AUC of 0.8 and a mean AUC of 0.92. When merging adjacent KL grades, all but one group showed near perfect results with AUC > 0.95 indicating excellent performance.

**Conclusion:**

We have found that we could teach a CNN to correctly diagnose and classify the severity of knee OA using the KL grading system without cleaning the input data from major visual disturbances such as implants and other pathologies.

## Background

With an aging population and increasing obesity worldwide, the prevalence of knee osteoarthritis (OA) is higher compared to other types of OA [1]. Knee arthroplasties in Sweden is expected to increase considerably the coming decade [2]. Similarly in the US, half of the population may have developed knee OA by the age of 85 [3] and the demand for total knee arthroplasty (TKA) is expected to be 6 times more common in 2030 as compared to 2005 [4]. Correctly diagnosing, classifying, following-up and planning for either conservative or operative management of knee OA is therefore of a great interest.

The diagnostic criteria of knee OA consist of a combination of pain, clinical and radiological findings. While pain is a key symptom, it is highly elusive and difficult to reliably quantify [5]. Radiographs in turn, correlate with number of symptoms [6] but there is a considerable discordance between radiographic findings and clinical presentation that is not fully understood [7]. Using MRI has been showing some promises, [8] but most orthopedic surgeons still rely on standard weight bearing radiographs. The Kellgren & Lawrence (KL) OA classification [9, 10] is a widely used grading system. By measuring joint space narrowing, osteophytic formations, subchondral sclerosis and then grading the severity from 0 to 4, radiologists would be able to assess the severity of the disease which could hint the surgeon as to further management. Improving the reliability and reproducibility of these interpretations could thus be hugely beneficial.

New technological advancement and recent progresses in medical image analysis using deep learning (DL), a form of artificial intelligence (AI) has been showing promising results in detecting knee OA and even classification of its severity based on the KL grading system [11–13]. Traditional machine learning (ML) has often put a lot of effort into extracting features before training the algorithms. With the DL network, on the other hand, we feed data directly to the algorithm and allow it to learn different features by itself. This has turned out to be a hugely successful approach which opens up new ways to non-experts in the field of ML to implement their own research and applications, such as medical image analysis [14]. To the best of our knowledge, there is only a few published articles on applying DL for classifying knee OA [11–13, 15–17]; However, these are mostly done, using pre-processed, highly optimized images.

The aim of this study was to develop and evaluate a neural network, using an entire radiographic series, without excluding common visual disturbances such as implants, casts, and other pathologies.

## Methods

### Study design and setting

This study is part of a model developed and validated as a diagnostic tool using our database containing radiographic examinations collected from our radiology department at Danderyd University Hospital. Recently, we have also published our results by Lind, et al. [18] based on this diagnostic method as it was able to use artificial intelligence to identify and classify fractures. The details of the source of data, extracting methods, neural network setup, outcome measures, and statistical analysis were identical with the previously published article [18].

We randomly selected radiographic images containing the knee that we divided into three sets: “training”, “validation”, and “test”. We excluded repeated knee exams for any individual with extra imaging within 90 days of the previous one to avoid overestimation by the network. In the test-set, the selection was intentionally biased towards OA, using text strings that the radiologists reported; hence, we were able to reduce the risk of non-OA cases to dominate the data. Trauma protocols (i.e. casts and fractures) as well as non-trauma protocols (implants, other pathologies) were included for the training data while the test data only included protocols that were marked as “weight bearing images of the knee “which is the standard method for evaluating OA. Diaphyseal femur and tibia/fibula protocols were also included as these display the knee joint although not in the center of the image. We excluded 0.6% of cases due to poor image quality as these could preclude classification. We have also excluded all radiographs of pediatric knees.

### Method of classification of OA

The primary outcome was both presence and severity of knee OA using the Kellgren & Lawrence (KL) osteoarthritis classification [9] which is a widely used knee OA classification system [10].

The outcome was established using a custom-built platform for labelling according to the KL grading scale by members of the research team (SO, AL, MG, EA). We also evaluated any potential OA features in the patellofemoral joint when lateral radiographs were also present. The lack of recognition of patellofemoral OA as a distinct or contributory factor according to the KL grading system has however been previously criticized [10]. We have also created custom output categories such as medial/lateral OA as it is interesting to see how well the network can discern these qualities on its own.

During training and model development, two sets of images were used. The training set which the network learned from and a validation set for evaluating performance and tweaking network parameters. The validation set was prepared in the same way as the test set but by SO and AL who are medical students. The training set was labeled only once by either SO or AL. If images were of bad quality or difficult to label, the students marked for revisit and were validated by MG. Initially, images were randomly selected for classification and fed to the network, i.e. passive learning. As the learning progressed, cases were selected based on the networks output, i.e. active learning [19]. Inclusion was stopped once performance stopped to improve by including more exams.

The test set was a separate set of examinations classified by two senior orthopedic surgeons, MG and EA, working independently. They had a joint reevaluation of conflicting cases until a consensus was reached. The test set then served as a ground truth for the final network to be tested against.

### Neural network setup

A supervised learning method was used to train a convolutional neural network of ResNet architecture [18–20]. We used this architecture because of its simplicity and lightweight with a total of 35 layers and a batch normalization for each convolutional layer as well as adaptive max pool (see Table 1). We trained the network initially without any noise for 100 epochs with a learning rate of 0.025 and then re-set the learning rate to 0.01 before training another 50 epochs with a combination of white noise (5%) and randomly erasing 3 blocks of 10 × 10 pixels.

**Table 1:** An overview of the network structure

Type	Blocks	Kernel Size	Filters	Group
ResNet block	1	3 × 3	64	Image
ResNet block	1	3 × 3	64	Image
ResNet block	6	3 × 3	64	Core
ResNet block	4	3 × 3	128	Core
ResNet block	2	3 × 3	256	Core
ResNet block	2	3 × 3	512	Core
Image max	1	–	–	Pool
Convolutional	1	1 × 1	72	Classification
Fully connected	1	1 × 1	4	Classification
Fully connected	1	1 × 1	4	Classification

We randomly initialized the network and trained using stochastic gradient descent and a cosine-function for the learning rate. During training we alternated between knee labels and other previously gathered fracture classification tasks (16,785 exams from other classification tasks [20]) where each task shared the core network.

### Input images

The network was presented with all available radiographs in each series. Each DICOM format radiograph was cropped using a separate OpenCV script to the active image area, i.e. any black border was removed, and the image was reduced to a maximum of 256 pixels. Image dimensions were retained by padding the rectangular image to a square format of 256 × 256 pixels. The images were additionally augmented during training with 2 jitters and separately processed up until a max pool merged the features into per image or exam depending on the type of outcome. In addition to the classification outputs that were pooled at the per exam level we had image view (i.e. AP, lateral, Oblique).

### Outcome measures/ statistical analysis

As previously presented by the authors [18] we measured the network performance primarily by using area under the curve (AUC). We have also used sensitivity, specificity and Youden J as secondary outcome measures. AUC of 0.7-0.8 is considered acceptable, 0.8-0.9 is considered good or very good and ≥ 0.9 is considered outstanding [21, 22]. Confusion matrices are presented to allow for good visualization of the algorithm’s performance when the true values are known. The network was implemented and trained using PyTorch (v. 1.4). Statistical analysis was performed using R (4.0.0).

## Results

### Outcome data

We included 5700 cases in the training set, 403 cases in the validation set and 300 cases in the test set. There was no patient overlap between the test and training datasets. The most common KL grade in the training and the test sets was KL grade 0 and KL grade 3, respectively. In the test set, KL grade 3 was the most common type, closely followed by grades 4 and 0. Implants were used as a major visual disturbance to put more stress on the DL network. 11% of cases in the training set and 20% of cases in the test set had some type of a visible implant (Table 2).

**Table 2:** Distributions between KL grades and implants in different data sets

	Train				Test			
	Yes		No		Yes		No	
	n	(%)	n	(%)	n	(%)	n	(%)
Kellgren-Lawrence								
0	2848	(50)	2852	(50)	67	(22)	233	(78)
1	1218	(21)	4482	(79)	23	(8)	277	(92)
2	652	(11)	5048	(89)	24	(8)	276	(92)
3	597	(10)	5103	(90)	116	(39)	184	(61)
4	380	(7)	5320	(93)	70	(23)	230	(77)
Location								
Medial	487	(9)	5213	(91)	120	(40)	180	(60)
Lateral	175	(3)	5525	(97)	34	(11)	266	(89)
Patella	36	(1)	2486	(44)	3	(1)	297	(99)
Implant	611	(11)	5089	(89)	61	(20)	239	(80)
TKA	226	(4)	5474	(96)	39	(13)	261	(87)
UKA	44	(1)	5656	(99)	7	(2)	293	(98)
Plate	111	(2)	5589	(98)	7	(2)	293	(98)
IM-nail	99	(2)	5601	(98)	0	(0)	300	(100)
IM-nail femur	68	(1)	5632	(99)				
IM-nail tibia	14	(0)	5686	(100)	0	(0)	300	(100)
Cerclage	55	(1)	5645	(99)	0	(0)	300	(100)
K-wires	21	(0)	5679	(100)	0	(0)	300	(100)
Staple	8	(0)	2514	(44)	4	(1)	296	(99)
X-fix	3	(0)	5697	(100)	0	(0)	300	(100)
Screws	57	(1)	5643	(99)	2	(1)	298	(99)
X-ligament	36	(1)	5664	(99)	5	(2)	295	(98)

*Abbreviations*: *TKA* Total knee arthroplasty, *UKA* Unicompartmental knee arthroplasty, *IM* intramedullary, *X-fix* external fixation, *X-ligament* signs of cruciate ligament reconstruction

### Network results

All five KL grades displayed good AUC > 0.80 with highest AUC for KL 0 with an AUC of 0.97, with sensitivity and specificity of 97 and 88%, respectively (Table 3). KL grade 2 had the lowest single performance with a sensitivity of 92%, specificity of 61% and an AUC of 0.80. When merging KL grades together generating larger groups, the network performed with AUCs of > 0.95 for all but the mid-ranged KL grade (KL 1, 2 and 3), which displayed an AUC of 0.82; suggesting that the classes in the middle cause most issues. For anatomical location, the network performed excellent in differencing between medial and lateral OA (Table 4).

**Table 3:** Network performance on outcome measures. Kellgren & Lawrence grades are displayed separately and merged

	Cases (*n* = 300)	Sensitivity (%)	Specificity (%)	Youden’s J	AUC (95% CI)
Kellgren-Lawrence					
0	67	97	88	0.85	0.97 (0.93 to 0.99)
1	23	96	75	0.70	0.88 (0.83 to 0.92)
2	24	92	61	0.53	0.80 (0.73 to 0.86)
3	116	92	71	0.63	0.87 (0.83 to 0.90)
4	70	84	78	0.63	0.87 (0.83 to 0.91)
Grouped Kellgren-Lawrence					
0 to 1	90	88	95	0.83	0.96 (0.94 to 0.98)
0 to 2	114	83	97	0.81	0.97 (0.95 to 0.98)
1 to 3	163	80	74	0.53	0.82 (0.77 to 0.87)
2 to 4	210	97	83	0.80	0.96 (0.94 to 0.98)
3 to 4	186	96	88	0.84	0.97 (0.95 to 0.99)

**Table 4:** Network performance on identifying medial or lateral OA

	Cases (n = 300)	Sensitivity (%)	Specificity (%)	Youden’s J	AUC (95% CI)
Lateral	34	100	89	0.89	0.97 (0.95 to 0.98)
Medial	120	91	88	0.79	0.94 (0.91 to 0.97)
Osteonecrosis	13	85	50	0.34	0.65 (0.54 to 0.75)
Patella	3	100	99	0.99	1.00 (0.99 to 1.00)

**Fig. 1:**
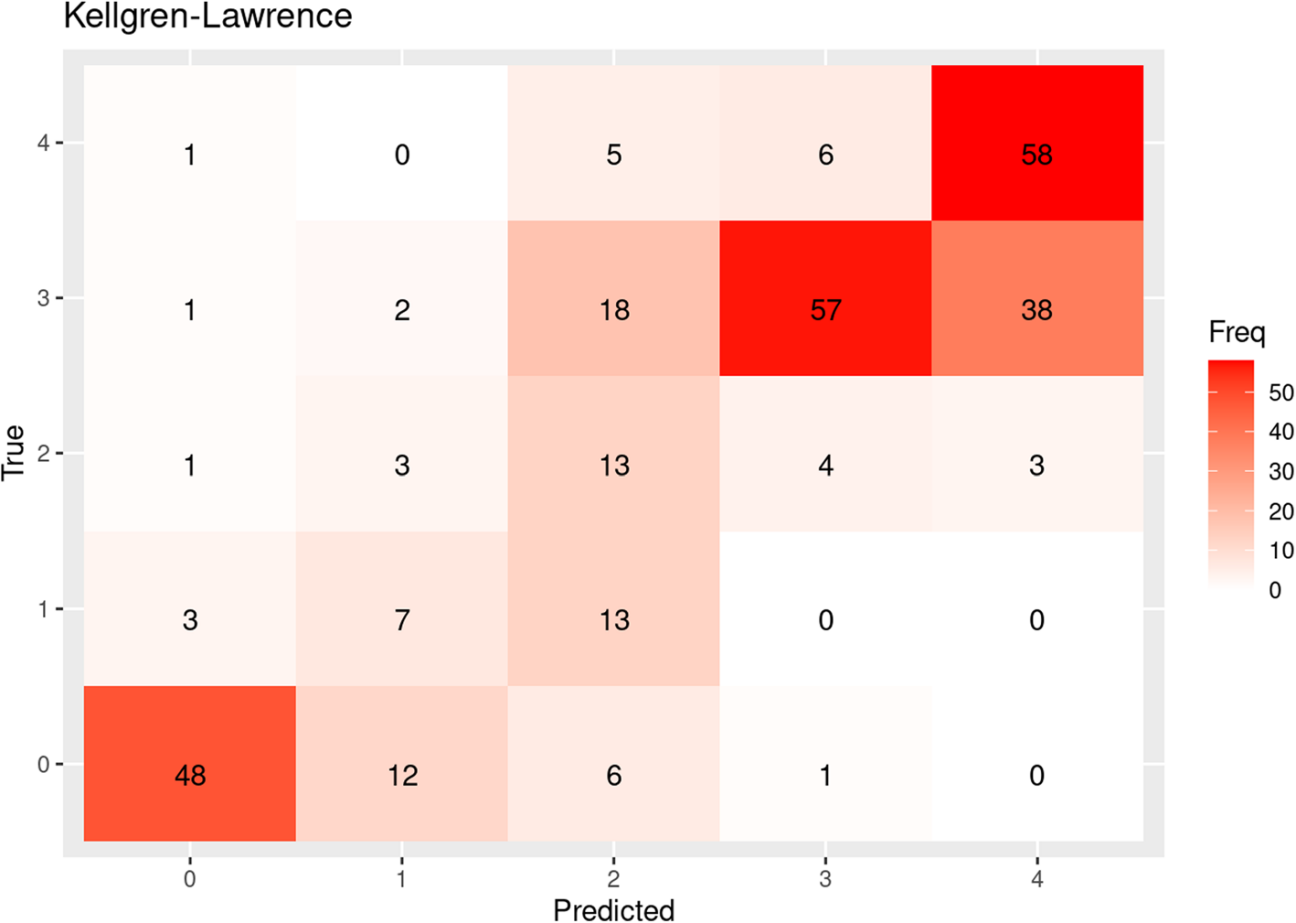
Confusion matrices between true label and predicted label in the test set. The numbers on X and Y- axis represents KL grades 0-4. Cases where both true and predicted labels match are seen diagonally.

The confusion matrices between the “true labels” (classified by senior orthopedic consultants) and “predicted labels” (by AI-network) demonstrate the ability of the DL network to classify the knee OA. As indicated by the AUC values, the network had most difficulties deciding whether to classify a knee OA as KL grade 1 or 2 (Fig. 1).

### Network decision analysis

**Fig. 2:**
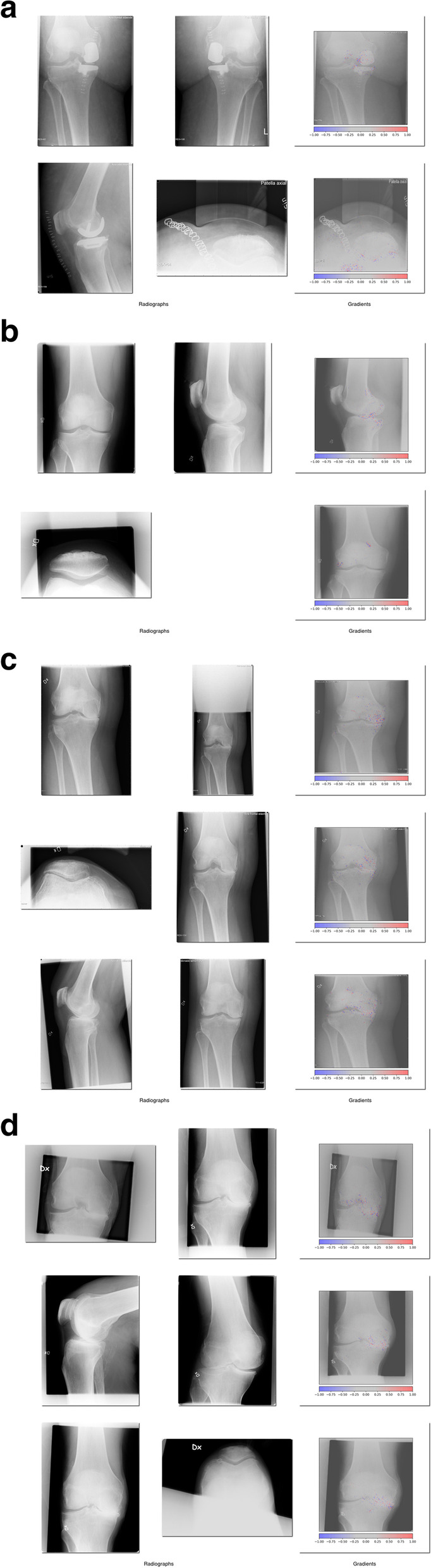
Examples from success and failure of the network. The integrated gradient heatmap shows red dots indicating features that contribute to the case being incorrectly classified, while blue dots indicate features weighing against it. **a**: Incorrectly classified KL grade 0. **b**: Correctly classified KL grade 1. **c**: Incorrectly classified KL grade 4. **d**: Correctly classified KL grade 4

We sampled cases for analysis where the network was most certain of a prediction, whether correct or incorrect. Examples of various KL grades are shown below. Heatmaps, visualizing areas in an image the network focuses on, are shown as colored dots, Fig. 2a to2d. There was no clear discernable trend explaining what made the network falter or succeed. In Fig. 2a as example, we see that the network suggests a class of 1-2 while the true class is 0. In this case, the heatmap activity is centered around the implant, where the network possibly reacts to remaining indicators of a previously operated medial arthrosis.

## Discussion

This study further demonstrates the potential of implementing AI-networks to aid in the diagnosis and even acceptably classifying knee OA. We believe this study, in accordance with other studies in the field, demonstrates DL potential in OA classification. Our network yielded an overall high AUC of more than 0.87 for all KL grades except KL grade 2 and a mean AUC of 0.92. When merging adjacent KL grades, all but one group showed near perfect results with AUC > 0.95. This further display that the network prediction is often close to the ground truth (as established by two orthopedic surgeons).

Network performance was generally excellent for all KL grades except for KL grade 2 which reached an AUC of 0.8. It is expected that images in the middle of the spectrum will be more difficult to assign proper category for, not only for DL networks but also for humans as these mid-categories have vaguer definitions. This is also demonstrated as we merged adjacent KL grades together; where all but mid-ranged KL group (KL 1, 2 and 3) showed near perfect results. We can also conclude that the AI- network was able to locate medial and lateral OA in the test set with a high precision.

The accuracy of our network is generally high compared to similar recent studies. As Swiecicki et al. [11] displayed in their 2021 paper, DL can now assess knee OA severity similar to radiologists. Tiulpin et al. [23] recently showed a new DL approach to significantly increase detection of radiographic OA presence. Mikhaylichenko et al. [12] paper have also shown promising results in applying different types of architecture in tackling knee OA grading, using the KL grading scale. Like our study, the lowest accuracy was in the mid-categories of the KL grading scale.

Major differences between our study and that of the above-mentioned ones are how we let the network learn from an entire image series without any preprocessing. Comparable studies often use big ImageNet datasets like that of “The Osteoarthritis Initiative (OAI)” [12, 15] or “Multicenter Osteoarthritis Study (MOST)” [11, 23] to train their networks. In contrast, all images used in our training set was taken from a general setting where radiographs can differ greatly in quality and even beam angels. We have also conducted, to the best of our knowledge, the first study letting the network learn from a whole image without manually locating the knee joint along with a high proportion of implants. Our study also shows the possibility of training and testing a DL network on a relatively small number of radiographs and still attaining a high AUC.

### Limitations

One limitation is the lack of different DL architecture tested. There is a vast amount of DL architectures and possibly some of them could have shown better results. It was, on the other hand, not our aim to find the superior DL architecture. Furthermore, the network can only be as good as the grading system it learns from. KL grading scale is the most used system for classifying knee OA. It is however, not a perfect grading system, with vague mid-category descriptions [10]. This makes ground truth being subjective to user preference, something that the DL network is not able to cope with. Prior studies [9, 24, 25] have reported that KL suffers from ambiguity with interobserver reliability. Culvenor et al. [26] made a study that compared knee OA using two different grading scales, KL and Osteoarthritis Research Society International (OARSI). They concluded that tibiofemoral OA was twice as common using OARSI grading scale compared to the KL system. There is a possibility we could have increased our accuracy of early osteoarthritis (grade 1-2) by using the OARSI system instead of KL.

While the radiographs used were collected from over a decade long period with a large sample of patients, our selection was limited in that the data source is a single hospital in Stockholm. From a generalization point of view, this study had disadvantages since all images were taken from PACS at Danderyd University Hospital with a mainly Caucasian population. The neural network would potentially present different results if tested on radiographs outside Sweden where ethnicity and joint alterations may differ.

### Clinical applications and future studies

As technology advances, so could expectation on classification accuracy. Further into the future, a possible task could be having the network calculate success of different treatment strategies, given features of the OA. It is however important to understand that OA is by nature a progressive disease with no clear boundary between KL grades. This consequently makes it impossible for a network to perform a perfect result. Correct application of a classification system for OA can nonetheless point towards the degree of severity and its progress alongside the clinical assessment of the patients, aiding physicians in evaluating the necessary treatment plans.

In a future study, it would be interesting to investigate other network architectures and computer vision algorithms. The current study uses a standard network and can function much as a baseline reference for future architectures. It would also be of interest to include patient’s symptoms and clinical signs in addition to radiographic findings in a DL network. This could later be used to analyze different orthopedic clinics in how keen they are to operate TKA-surgery on different KL grades. As MRI becomes more available and cheaper, a shift from weight-bearing plain radiographs to MRI would make studies between different modalities and DL noteworthy as well.

## Conclusion

We found that we could teach a neural network to classify knee OA severity and laterality using the KL grading scale without cleaning the input data from major visual disturbances such as implants and other pathologies.

## Data Availability

The test dataset used during evaluation phase of the current study available from https://datahub.aida.scilifelab.se/10.23698/aida/koa2021 and the code is available at https://github.com/AliRazavian/TU.

## Abbreviations

AI: Artificial intelligence
AO: Arbeitsgemeinschaft für Osteosynthesefragen
AP: Anteroposterior
AUC: Area under the ROC Curve
CNN: Convolutional neural network
DICOM: Dig ital Imaging and Communications in Medicine
DL: Deep learning
IM: Intramedullary nail
KL: Kellgren & Lawrence grading scale
ML: Machine learning
MOST: Multicenter Osteoarthritis Study
MRI: Magnetic resonance imaging
OA: Osteoarthritis
OAI: The Osteoarthritis Initiative
OARSI: Osteoarthritis Research Society International
PACS: Picture archiving and communication system
ROC: Receiver Operating Characteristic
ResNet: Residual network
TKA: Total knee arthroplasty
UKA: Unicompartmental knee arthroplasty
US: United States
X-fix: External fixation
X-ligament: Cruciate ligament reconstruction
